# Size and Treatment Outcomes of HR+, HER2- Early Breast Cancer Population With High Risk of Recurrence: A Real-World Cohort Study With Danish Breast Cancer Cooperative Group Registry Data

**DOI:** 10.36469/001c.137277

**Published:** 2025-06-24

**Authors:** Heidi Loponen, Juha Mehtälä, Laila Mehkri, Astrid Torstensson, Anna Emde, Tero Ylisaukko-oja, Walid Fakhouri

**Affiliations:** 1 MedEngine Oy, Helsinki, Finland; 2 MedEngine DK Aps, Copenhagen, Denmark; 3 Eli Lilly Sweden Ab, Solna, Sweden; 4 Eli Lilly and Company, Indianapolis, Indiana, USA

**Keywords:** early breast cancer, high risk of recurrence, patient characteristics, survival, HR+/HER2-

## Abstract

**Background:** While the prognosis is generally good for hormone receptor–positive (HR+), human epidermal growth factor–negative (HER2–) early breast cancer (EBC) patients, up to 30% of patients with high-risk clinical and/or pathologic features experience recurrence. **Objectives:** This retrospective cohort study was designed to estimate the proportion of BC patients meeting the high-risk criteria used in monarchE, a phase III study of abemaciclib, and to describe the characteristics, survival, and disease recurrence in a Danish patient population. **Methods:** The study cohort included all women with BC diagnosis registered in the Danish Breast Cancer Cooperative Group registry, and lumpectomy or mastectomy performed between January 1, 2010, and December 31, 2019. The patient characteristics and survival outcomes were compared between high-risk patients (≥4 positive lymph nodes or 1-3 positive nodes and grade 3 and/or primary tumor size ≥5 cm), low/moderate-risk patients, and patients with triple-negative EBC (TNBC). **Results:** A total of 13.0% of the HR+, HER2– EBC patients met the high-risk criteria. Five-year invasive disease-free survival (IDFS) and distant recurrence-free survival rates (DRFS) were significantly lower in the high-risk group (73.9% and 75.9%, respectively) and the TNBC group (73.0% and 76.5%, respectively), than the low/moderate-risk group (86.1% and 87.7%, respectively) (P < .0001). **Discussion:** This study is in line with earlier observations showing that HR+, HER2– is the most common subtype, accounting for over 70% of all BC cases. The size of the monarchE-like high-risk group aligns with previous evidence from large US cohort studies. We observed that the proportion of TNBC among all EBC patients showed a decreasing trend between 2010-2019, consistent with earlier reports. The 5-year IDFS and DRFS rates of high-risk patients observed in this study are in line with the evidence from a large US cohort study, however, slightly lower IDFS and DRFS rates at 5 years for the low/moderate-risk group were observed here. **Conclusion:** About 13.0% of the HR+, HER2– EBC patient population has a high risk of recurrence and would likely benefit from novel treatment strategies targeted for patients with a high risk of recurrence.

## BACKGROUND

Breast cancer (BC) is the most frequently diagnosed cancer in women. Between 2016 and 2020, BC affected about 7.8 million women globally, with an estimated annual incidence of 55.2 per 100 000 and an age-standardized mortality rate of 14.9 per 100 000.^1,2^ In Denmark, in 2020, there were 5083 new cases, 1121 deaths, and 21 879 prevalent BC cases among all ages.^3^ From 2016 to 2020, 1- and 5-year survival rates for Danish patients with BC were 97.4% and 90.2%, respectively.^4^

Over 90% of patients with BC in high-income countries are diagnosed with early-stage disease (ie, early breast cancer [EBC]), although significant regional variations are observed.^5,6^ The most prevalent subtype of BC is hormone receptor–positive (HR+) human epidermal growth factor receptor 2–negative (HER2–), accounting for over 70% of all BCs.^6–9^ Treating EBC is complex and involves a combination of local modalities (surgery, radiotherapy [RT]), systemic anticancer treatments (chemotherapy [CT], endocrine therapy [ET], targeted molecular therapies) and supportive measures based on patient characteristics, tumor burden/location, and pathology.^10,11^ Guidelines recommend that following primary treatment (including surgical excision), adjuvant ET should be considered for all HR+ tumors since this has been associated with a significant reduction in the risk of recurrence and death.^12^

While the prognosis for HR+, HER2– EBC is generally favorable, recurrence remains a concern. Approximately 20% of patients experience recurrence within 10 years, often involving distant metastases that render the disease incurable. Over time, this figure increases to around 30% of patients developing locoregional or distant recurrence.^13–15^ For patients with high-risk clinical and/or pathological factors, the likelihood of recurrence is increased, particularly during the initial years of adjuvant ET.^16^ In HR+ BC patients, around 50% of recurrences occur within the first 5 years, indicating resistance to ET,^17^ and early recurrences in HR+, HER2– EBC patients peaking in the first years on ET.^18,19^ Thus, it is essential to optimize adjuvant therapy to prevent early recurrences and metastases in these patients.^20^

Despite well-recognized individual clinicopathological risk factors for recurrence, such as the presence of positive axillary lymph nodes (ALN) and higher disease stage, a standard definition for high risk has not been formally established.^18,19^ Thus, there is no standardized approach for identifying and targeting high-risk patient groups. This is important since high-risk patients can potentially benefit from the addition of new adjuvant treatments to current multimodal therapy. Novel treatment strategies investigated include cyclin-dependent kinase (CDK) 4 (CDK4) and CDK6 inhibitors, selective estrogen receptor degraders (SERD), phosphoinositide 3-kinase (PI3K) inhibitors, mammalian target of rapamycin (mTOR) inhibitors, and poly (ADP-ribose) polymerase (PARP) inhibitors.^20–24^

Abemaciclib is an orally administered, continuously dosed CDK4/6 inhibitor. In 2022, the European Medicines Agency approved it for use in combination with ET for the adjuvant treatment of adult patients with HR+, HER2–, node-positive EBC at high risk of recurrence. Additionally, premenopausal or perimenopausal women receiving aromatase inhibitor ET should also be given a luteinizing hormone–releasing hormone agonist.^25^ Per EU labeling, the high risk of recurrence is defined as the presence of ≥4 positive ALNs or 1 to 3 positive ALNs and histologic grade 3 and/or tumor size ≥5 cm.^25^ The results from the monarchE trial showed that abemaciclib, when added to ET, significantly improved invasive disease-free survival (IDFS) in patients with high-risk EBC in the adjuvant setting.^20,26,27^ There are only a few studies in the real-world clinical setting on the abemaciclib target population. The largest real-world cohort studies published to date have used patient data from the United States (US), but additional data from Europe are required. In a nationwide real-world study in the US, BC patients at high risk of recurrence were identified based on the monarchE trial criteria (≥4 positive ALNs or 1-3 positive ALNs and grade 3, tumor size ≥5 cm, and/or Ki-67 ≥20%). Patients with high-risk features accounted for 13.8% of all HR+, HER2– EBC patients. The risk of recurrence or death was 3 times higher in patients with tumors that exhibited high-risk features than in those in the non-high-risk group.^28^ Similarly, in another US study where NIH Surveillance, Epidemiology, and End Results Program (SEER) EBC patient data were used, 12.0% of all HR+, HER2– EBC cases were classified as being at increased risk of recurrence (using monarchE/Cohort 1 criteria of ≥4 positive ALNs or 1-3 positive ALNs and histologic grade 3 and/or tumor size ≥5 cm). They also found that histologic grade 3 was the most influential factor on mortality among the HR+, HER2– EBC patients.^29^

In addition to the HR+, HER2– high-risk BC patient population, triple-negative BC (ER–, PR–, HER2–)(TNBC) represents a highly aggressive type of BC characterized by poorer prognosis and an unmet medical need for improving adjuvant systemic therapy.^30,31^ The 5-year survival rates described for TNBC in previous literature vary between 54% and 79.6%, a life expectancy of 3.55 years has been estimated, and having ≥4 ipsilateral ALN was found to be an influential factor on mortality of these patients.^29,32–36^ Kohler et al reported a 5-year survival rate of 65% for TNBC patients presenting regional tumors and 11% for cases where the tumor has spread to distant organs.^32^

The primary aim of this study was to estimate the proportion of BC patients meeting the high-risk criteria used in the monarchE trial of abemaciclib in the adjuvant setting to provide yet unavailable real-world evidence from a large European cohort. Hence, our study cohort was formed from the Danish Breast Cancer Cooperative Group (DBCG) registry with almost nationwide coverage (98.4%).^37,38^ In addition, we aimed to describe the characteristics, survival, and disease recurrence in the high-risk, low/moderate-risk (HR+, HER2– EBC not meeting the monarchE high-risk definition), and TNBC patients.

## METHODS

This study was a non-interventional, retrospective, registry-based cohort study. The study cohort included women (>18 years) with a BC diagnosis (*International Classification of Diseases, Tenth Revision* [ICD-10]: C50) in the DBCG registry, and lumpectomy or mastectomy performed between January 1, 2010, and December 31, 2019. Patients with metastatic disease or other primary malignancy were excluded from the cohort. Patients were followed up until the date of disease recurrence, death, emigration, or December 31, 2020, whichever occurred first. Data were retrospectively collected from the DBCG database, the Danish National Patient Registry, the Danish Pathology Register, the Danish Civil Registration System, and the Cause of Death Register. The DBCG database, established in 1977, is a comprehensive, population-based registry capturing nearly all BC cases in Denmark through mandatory reporting. It contains detailed data on patient demographics, tumor characteristics, treatments (surgery, RT, systemic therapy), and outcomes, enabling longitudinal follow-up.

The following variables were collected: age at BC diagnosis (years), menopausal status (pre/post), tumor size (<2 cm; >2 cm to <5 cm; >5 cm), number of positive lymph nodes (0/1 to 3/>4), histologic grade (1/2/3), Ki-67 index (%), HER2 status (+/–), estrogen receptor status (<1%/≥1%), type and date of surgery (mastectomy/lumpectomy), comorbidities (ICD-10), family history of BC (ICD-10: Z80.3), date and status at follow-up, date of emigration, date of death, and treatment type (RT/CT/ET). To assess the number of comorbidities, the following diagnoses were included: myocardial infarction (ICD-10: I21*, I22*, I23*), congestive heart failure (I50.0), peripheral vascular disease (I73*-I79*), cerebrovascular disease (I60*-I69*), hemiplegia or paraplegia/tetraplegia (G81*, G82*), dementia (F00*–F03*), chronic pulmonary disease (J40*-J47*), rheumatologic disease (M05, M32, M33, M34, M06, M31.5, M35.1, M35.3, M36.0), peptic ulcer disease (K25*-K28*), diabetes (E10*-E14*), renal disease (failure) (N17*-N19*), liver disease (K70*-K77*), AIDS/HIV (B20*-B24*), hypertension (I10*-I15*), and dyslipidemia (E78*).

### Patient Subgroups

Patients were classified into subgroups according to the American Joint Committee on Cancer TNM classification system for malignant tumors (8th edition) and tumor marker profile (HER2 and HR status).^39^ Patients with HR+, HER2– EBC with ≥4 positive ALNs or 1 to 3 positive ALNs and grade 3 and/or primary tumor size ≥5 cm (monarchE/Cohort 1 definition) were classified as patients with a high risk of recurrence (high-risk group). Patients with HR+, HER2–, node-positive EBC (stage IB-IIIC) not meeting the abovementioned criteria were classified into the low/moderate-risk group. Patients with HR–, HER2– EBC (IA-IIIC) were categorized into the TNBC group (**Supplemental Table S1**).

### Outcome Measures

IDFS was measured from the date of surgery to the date of the first occurrence of the following events: ipsilateral, regional, or distant recurrence, death from any cause, contralateral invasive BC, and second primary non-breast invasive cancer.^40^ Distant recurrence-free survival (DRFS) was defined as the time from the date of surgery to distant recurrence or death from any cause, whichever occurred first. Overall survival (OS) was defined as the time from the date of surgery to the date of death from any cause.

### Statistical Analyses

The number of incident cases in different groups in the DBCG registry was estimated in the yearly cohorts (2010-2019) and overall. The estimates with a 95% confidence interval (CI) were derived from a Poisson distribution.

Descriptive analyses were performed to assess the clinical characteristics. Medians with first and third quartiles (Q1, Q3) were described for continuous variables. An independent Wilcoxon test was used to determine the difference between groups. Frequencies and proportions were used for categorical variables. The differences were determined by the ξ^2^ test (significance level, 95%), and the standardized mean differences (SMDs) were calculated. Only existing data were utilized, and no missing values were imputed.

OS, IDFS, and DRFS were described using the Kaplan-Meier method and compared using the log-rank test. CIs for the Kaplan-Meier estimates were acquired using the Greenwood formula applied with the log-transformation method. These outcomes were assessed from the most recent 5-year period of the study (2015-2019) and for the whole study period. In addition, the Cox regression with independent variables family history, type of surgery, histologic grade, TNM stage, menopausal status, number of comorbidities, and age at diagnosis with Wald test for significance were used for further adjusted analyses.

## RESULTS

### Proportion of High-Risk, Low/Moderate-Risk, and TNBC Patients

In total, 33 631 patients were included in the study cohort (stage 0-IIIC, including occult stage patients), of which 30 482 had EBC. The number of patients with HR+, HER2– EBC was 23 813. Of the EBC patients, 3098 were classified as high-risk patients, 5308 as low/moderate-risk patients, and 2520 as TNBC patients (**Table 1**). Between 2010 and 2019, the overall proportion of high-risk patients out of all EBC patients was 10.2% (95% CI: 9.8-10.5). The corresponding figures for low/moderate-risk and TNBC groups were 17.4% (16.9-17.9) and 8.3% (7.9-8.6), respectively. A slight decrease in the proportion was observed in the high-risk group after 2016 (11.2% in 2016 vs 9.0% in 2019), and in the TNBC group after 2015 (8.5% in 2015 vs 6.5% in 2019) (**Table 1**). The overall proportion of high-risk patients out of the HR+, HER2– EBC population was 13.0%.

**Table 1.** Proportion of High-Risk, Low/Moderate-Risk, and TNBC Patients Among All EBC Patients Yearly (2010-2019) and Overall

**Year**	**High Risk (n)**	**Low/Moderate Risk (n)**	****TNBC** **(n)****	**All With EBC (N)**	**Proportion High Risk (95% CI)**	**Proportion Low/Moderate Risk (95% CI)**	**Proportion TNBC (95% CI)**
2010	385	689	293	3599	10.7 (9.7-11.8)	19.1 (17.8-20.6)	8.1 (7.2-9.1)
2011	333	560	271	3171	10.5 (9.4-11.7)	17.7 (16.2-19.2)	8.5 (7.6-9.6)
2012	320	545	324	3110	10.3 (9.2-11.5)	17.5 (16.1-19.0)	10.4 (9.3-11.6)
2013	334	537	288	3205	10.4 (9.3-11.6)	16.8 (15.4-18.2)	9.0 (8.0-10.1)
2014	317	575	281	3258	9.7 (8.7-10.8)	17.6 (16.2-19.1)	8.6 (7.7-9.7)
2015	319	536	261	3083	10.3 (9.3-11.5)	17.4 (16.0-18.9)	8.5 (7.5-9.5)
2016	330	489	228	2950	11.2 (10.0-12.4)	16.6 (15.2-18.1)	7.7 (6.8-8.8)
2017	273	470	202	2804	9.7 (8.6-10.9)	16.8 (15.3-18.3)	7.2 (6.3-8.2)
2018	252	459	202	2702	9.3 (8.2-10.5)	17.0 (15.5-18.6)	7.5 (6.5-8.6)
2019	235	448	170	2600	9.0 (7.9-10.2)	17.2 (15.7-18.9)	6.5 (5.6-7.6)
Total	3098	5308	2520	30 482	10.2 (9.8-10.5)	17.4 (16.9-17.9)	8.3 (7.9-8.6)

Abbreviations: CI, confidence interval; EBC, early breast cancer; TNBC, triple-negative breast cancer.

### Patient Characteristics

Patient characteristics for high-risk, low/moderate-risk, and TNBC patients are presented in **Table 2**. The median age (Q1, Q3) at diagnosis was 63.8 years (52.2, 73.6) in the high-risk group, 63.0 (53.0, 71.1) in the low/moderate-risk group, and 61.8 (50.0, 71.1) in the TNBC group. A statistically significant difference was seen between the high-risk group and TNBC group (*P* < .001; SMD, 0.155). A higher proportion of patients in all 3 groups was postmenopausal (72.0%-76.9%) than premenopausal (23.1%-28.0%).

**Table 2.** Patient Characteristics in High-Risk, Low/Moderate-Risk, and TNBC Patients

**Variable**	**High Risk**	**Low/Moderate Risk**	*****P*** **Value****	**SMD**	**TNBC**	*****P*** **Value****	**SMD**
N	3098	5308			2520		
Follow-up length (y), median (Q1, Q3)	4.40 (2.30, 6.56)	4.99 (2.77, 7.20)	< .001	0.181	3.53 (1.62, 6.13)	< .001	0.182
Age at diagnosis (y), median (Q1, Q3)	63.8 (52.2, 73.6)	63.0 (53.0, 71.1)	.043	0.029	61.8 (50.0, 71.1)	< .001	0.155
Menopausal status, n (%)							
Pre	781 (25.2)	1227 (23.1)	.032	0.049	705 (28.0)	.021	0.063
Post	2317 (74.8)	4081 (76.9)			1815 (72.0)		
Missing	0	0			0		
Tumor size, n (%)							
<2	870 (28.2)	3044 (59.6)	< .001	0.848	1268 (50.9)	< .001	0.571
≥2 to <5	1686 (54.7)	2060 (40.4)			1114 (44.7)		
≥5	526 (17.1)	0			108 (4.3)		
Missing	16	204			30		
No. (%) of positive lymph nodes							
0	0 (0.0)	0 (0.0)	NA	1.773	1733 (68.8)	< .001	2.262
1-3	1205 (38.9)	5308 (100.0)			564 (22.4)		
≥4	1893 (61.1)	0 (0.0)			223 (8.8)		
Missing	0	0			0		
Histologic grade, n (%)							
1	373 (12.2)	1799 (35.2)	< .001	1.370	28 (1.3)	< .001	0.775
2	1239 (40.6)	3310 (64.8)			402 (18.2)		
3	1439 (47.2)	0 (0.0)			1776 (80.5)		
Missing	47	199			314		
Ki-67, n (%), median (Q1, Q3)	30.0 (15.0, 50.0)	15.0 (10.0, 25.0)	< .001	0.742	70.0 (50.0, 90.0)	< .001	1.167
Missing	921	1698			787		
Type of surgery (%)							
Lumpectomy	1387 (44.8)	3556 (67.0)	< .001	0.460	1742 (69.3)	< .001	0.515
Mastectomy	1703 (55.0)	1741 (32.8)			763 (30.4)		
Missing	8	11			15		
No. (%) of comorbidities							
0	1994 (64.4)	3565 (67.2)	< .001	0.091	1630 (64.7)	.626	0.026
1	637 (20.6)	1106 (20.8)			532 (21.1)		
≥2	467 (15.1)	637 (12.0)			358 (14.2)		
Missing	0	0			0		
Treatment type, n (%)							
ET (±CT)	2957 (99.0)	5147 (99.6)	.010	0.065	0 (0.0)	< .001	14.566
CT only	11 (0.4)	6 (0.1)	.031	0.051	2249 (92.4)	< .001	4.800
No	18 (0.6)	17 (0.3)			184 (7.3)		
Missing	112	138			87		
Radiation therapy, n (%)							
Yes	2509 (87.2)	4369 (88.5)	.102	0.039	1872 (92.9)	< .001	0.188
No	367 (12.8)	567 (11.5)			144 (7.1)		
Missing	222	372			504		

Abbreviations: CT, chemotherapy; ET, endocrine therapy; SMD, standardized mean difference; TNBC, triple-negative breast cancer.

*P* values and SMD are presented relative to the high-risk group.

In the high-risk group, a significantly larger proportion of patients had tumor sizes ranging from ≥2 to <5 cm (54.7%) or ≥5 cm (17.1%) than low/moderate-risk (40.4% and 0.0%, respectively) and TNBC groups (44.7% and 4.3%, respectively) (*P* < .001). As per the selection criteria of the low/moderate-risk group, all patients had 1 to 3 positive lymph nodes, and a histologic grade of 1 (35.2%) or 2 (64.8%). In the high-risk group, 38.9% had 1 to 3 positive lymph nodes, and 61.1% had ≥4 positive nodes. Most patients in the TNBC group were node-negative (68.8%); and had histologic grade 3 tumors (80.5%). The median Ki-67 index was the highest in the TNBC group (70.0%; Q1, Q3: 50.0%, 70.0%). The corresponding numbers in the high-risk and in the low/moderate-risk groups were 30.0% (Q1, Q3: 15.0, 50.0%) and 15.0% (Q1, Q3: 10.0, 25.0%), respectively.

In the high-risk group, mastectomy was performed for 55.0% of the patients. In the low/moderate-risk group and TNBC group, the corresponding percentages were 32.8% and 30.4%, respectively.

In the high-risk and low/moderate-risk groups, 99.0% and 99.6% of the patients received ET, respectively. Most patients in the TNBC group (92.4%) received CT. In addition to systemic treatment, around 90% of patients in all 3 groups also received RT.

### Breast Cancer Recurrence and Overall Survival

**Figure 1.**
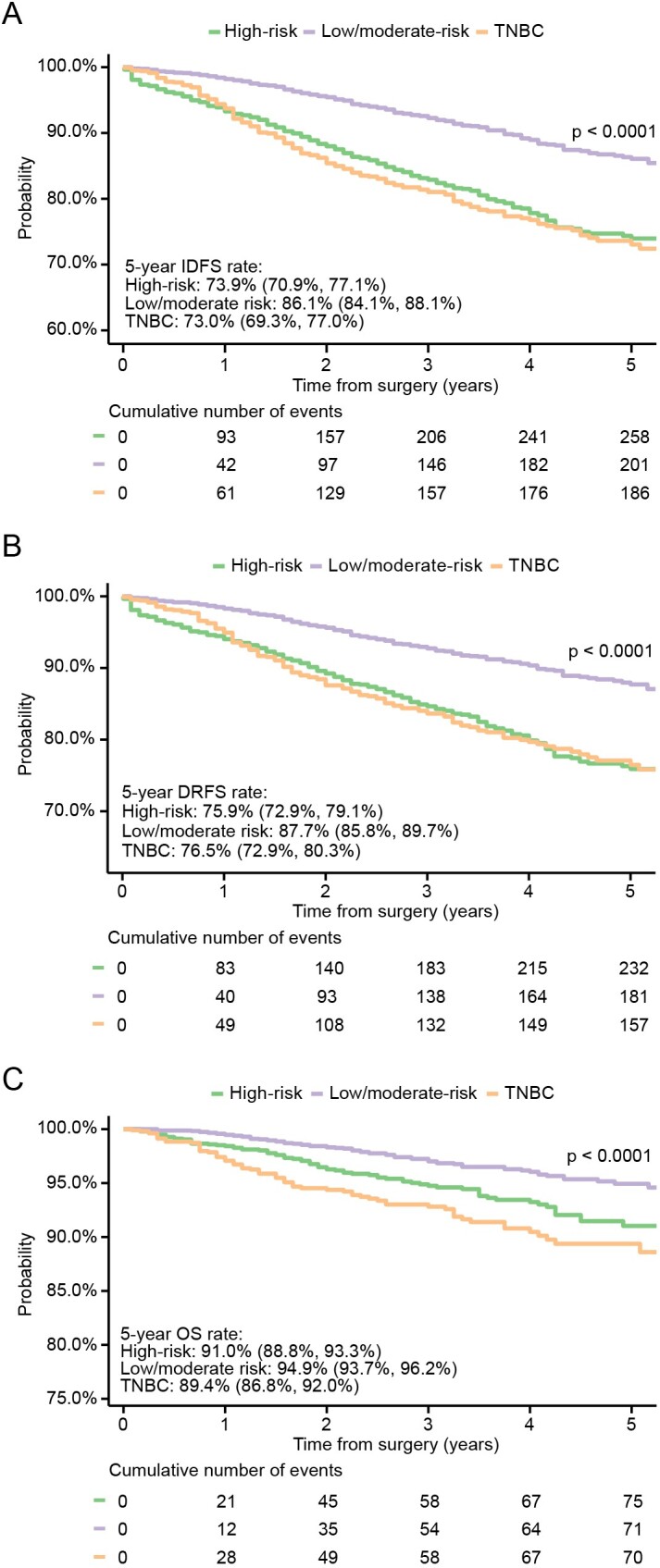
(**A**) Invasive Disease-Free Survival, (**B**) Distant Recurrence–Free Survival, and (**C**) Overall Survival During 2015-2019 in High-Risk, Low/Moderate-Risk, and Triple-Negative Breast Cancer Patients Abbreviations: DRFS, distant recurrence–free survival; IDFS, invasive disease–free survival; OS, overall survival; TNBC, triple-negative breast cancer.

IDFS, DRFS, and OS were assessed and compared between the 3 groups from the most recent 5-year period of the study (2015 to 2019). For the high-risk group, the IDFS rate at 5 years was 73.9% (95% CI: 70.9%-77.1%). In the low/moderate-risk and TNBC groups, the corresponding rates were 86.1% (84.1%-88.1%) and 73.0% (69.3%-77.0%), respectively. Rates of DRFS at 5 years were 75.9% (72.9%-79.1%), 87.7% (85.8%-89.7%), and 76.5% (72.9%-80.3%) for the high-risk, low/moderate-risk, and TNBC groups, respectively. OS rates at 5 years were 91.0% (88.8%-93.3%), 94.9% (93.7%-96.2%), and 89.4% (86.8%-92.0%) for the high-risk, low/moderate-risk, and TNBC groups, respectively (**Figure 1**). When OS was assessed for the whole study period, the 10-year OS rates were 80.7% (76.2%-85.4%), 89.3% (87.6%-91.0%), and 86.8% (84.7%-88.9%) for the high-risk, low/moderate-risk, and TNBC groups, respectively (*P* < .0001; data not shown).

When assessed with the Cox multivariate model in the high-risk group, the risk of having an IDFS event was highest among those aged 39 years or younger and 70 years or older (**Supplemental Figure S1**). The risk also increased with higher number of comorbidities, advancing TNM stage, and higher histologic grade (*P* < .001). Regarding DRFS (**Supplemental Figure S2**), similar associations were seen with the number of comorbidities, TNM stage, histological grade (all *P* < .001), and age (*P* < .01). In addition, mastectomy as type of surgery was associated with an increased risk for DRFS (*P* < .05). For death (**Supplemental Figure S3**), the number of comorbidities, advancing TNM stage (*P* < .001), higher histological grade, and mastectomy as surgery type (*P* < .01) again increased the risk. Among age groups, highest risk was observed in those aged 70 years or older (*P* < .01).

## DISCUSSION

For nearly 20 years, adjuvant ET has been a standard treatment of HR+, HER2– EBC and is associated with a significant reduction in risk of recurrence and death.^11^ However, up to 30% of patients receiving adjuvant ET with high-risk clinical and/or pathologic features experience recurrence.^16^ Thus, there is a clear unmet medical need for this group of patients. In 2022, abemaciclib was approved for the adjuvant treatment of HR+, HER2–, node-positive EBC at high risk of recurrence.^20^ In the clinical trial setting, the addition of abemaciclib to ET resulted in a 25% reduction in the risk of developing an IDFS event relative to ET alone and a 3.5% absolute improvement in 2-year IDFS rate after a median follow-up of 15.5 months (92.2% vs 88.7%).^20^ Further follow-up (median, 42 months) showed a sustained treatment effect with abemaciclib: 33.6% reduction in IDFS events and a 6.4% absolute risk-difference at 4 years (85.8% vs 79.4%).^26^ Patients with a high Ki-67 index were shown to have worse outcomes, validating Ki-67 as a prognostic marker. However, the benefit with abemaciclib was consistent regardless of the Ki-67 index: the redsuction in the risk of developing an IDFS event relative to ET alone was 37% in the Ki-67–high population and 30% in the Ki-67–low population.^27^

This registry-based cohort study was primarily designed to estimate the size of the EBC population meeting the high-risk criteria used in the monarchE trial in a European real-world setting, adding to the available findings on this subpopulation.

In total, 33 631 BC patients (stage 0-IIIC) were included in this study, of which 30 482 (90.6%) had EBC (stage IA-IIIC). The number of patients with HR+, HER2– EBC was 23 813, which corresponds to 78.1% of all EBC patients, and the number of patients with TNBC was 2520 (8.3% of all EBC patients). This is in line with global cancer statistics and previous real-world cohort studies showing that HR+, HER2– is the most common subtype, accounting for over 70% of all new BC cases.^2,8,41–44^ Overall, the reported incidence rates of TNBC vary between 10% and 20% in all new BC cases, although some European studies have reported rates under 10%.^8,30–32,42–44^ Consistent with an earlier report by Andersson et al, we observed that the proportion of TNBC among all EBC patients showed a decreasing trend during the study period of 2010 to 2019.^41^

A recent US real-world study using a nationwide Flatiron Health electronic health record–derived database assessed the size of the monarchE-like high-risk group. Of the stage IA–IIIC HR+, HER2– patients, 13.8% were defined as high-risk patients, which corresponds well to the size of the high-risk group in this study (10.2% of all EBC patients, and 13.0% of the HR+, HER2– EBC patients). The proportion of the low/moderate-risk group was relatively stable during the study period, and the proportion of the high-risk group even showed a slight decline during the most recent years. Of note, the US study used slightly different criteria for defining the high-risk group by also including Ki-67 of ≥20% as an alternative criterion for patients with 1 to 3 positive lymph nodes.^28^ Similarly, in another real-world US study utilizing SEER EBC patient data, 12.0% of all HR+, HER2– EBC patients were included in the high-risk group as per monarchE/Cohort 1 criteria.^29^ Conversely, in a German single-center and in a French cross-sectional real-world study, higher proportions of 19.4% and 26.2% of EBC patients with a HR+, HER2− subtype, fulfilled the monarchE high risk criteria.^44,45^ This difference might be because these studies had rather small cohorts, even from a single center, which are more prone to selection bias.

The nationwide US real-world study using Flatiron data showed that the 5-year IDFS rate was 70.2% in the high-risk group and 90.9% in the non-high-risk group (*P* < .0001). Regarding the DRFS, the rates were 71.9% and 91.8% for the high-risk and non-high-risk groups, respectively.^28^ The 5-year IDFS and DRFS rates of high-risk patients observed in this study (73.9% and 75.9%, respectively) are in line with the recent US observations. We observed slightly lower IDFS and DRFS rates at 5 years for the low/moderate-risk group (86.1% and 87.7%, respectively), compared with the non-high-risk group in the US/Flatiron cohort, which could be because the US cohort also included node-negative patients in the non-high-risk group. The 2-year IDFS rate reported for the high-risk group in the US study (88.1%) was consistent with the 2-year IDFS rate for the ET-alone arm in the monarchE trial (90.0%).^20,28^

The US/Flatiron study did not report OS data, and the OS data from the monarchE trial remained immature as of the latest follow-up.^26,28^ In the other US study using SEER data, the 5-year mortality rate for high-risk patients was 16.5%, and 7.0% for those who did not meet the high-risk criteria.^29^ In this study, the 5-year OS rates (2015-2019) were 91.0% and 94.9% for the high-risk and low/moderate-risk groups, respectively, and 10-year rates (during the whole study period) were 80.7% and 89.3%, respectively, showing a greater absolute difference between the groups at longer follow-up. According to the SEER statistics, the 5-year OS rate in the HR+, HER2– subtype is 94.4% when all stages are combined. For localized disease, the OS rate is 100.0%, and for regional disease 91.1%.^42^ The latest Nordcan statistics (2016-2020) showed a 5-year OS rate of 90.2% (95% CI: 89.5%-90.9%) for total Danish BC population, in line with our above-mentioned observations.^4^ The definition of high recurrence risk has varied over time in DBCG treatment programs, reflecting the absence of a standard criterion. Frequent updates to treatment recommendations further limit the comparability of survival outcomes across earlier studies on high-risk patient populations.^37,46,47^

The characteristics of the high-risk cohort in this study were very similar to those observed in the corresponding US, French, and German cohorts, but certain differences were observed compared with the patients of the ET arm in the monarchE trial. The median age at diagnosis was 63.8 years in the high-risk cohort in our study, close to what has been observed in the former real-world studies (58.5-60.2 years).^28,29,44,45^ These are noticeably higher than in the monarchE trial (51 years). The proportion of patients having 1 to 3 positive nodes was similar between our high-risk cohort (38.9%) and the monarchE trial cohort (40.4%).^26^ Likewise, in the monarchE trial cohort, tumor sizes <2 cm and ≥5 cm were fairly evenly distributed (27.1% vs 21.6%, respectively), but in our high-risk cohort, tumor size <2 cm dominated over ≥5 cm (28.2% vs 17.1%, respectively). In total, 47.2% of the high-risk patients in this study had histologic grade 3, and the corresponding proportions in the monarchE trial and in the former real-world cohort studies were 37.6% and 43.3% to 55.2%, respectively.^26,28,44,45^

Compared with other subtypes of BC, TNBC is more prone to recurrence and metastasis and has a poorer prognosis. In the current study, the TNBC group showed inferior survival outcomes in all endpoints assessed, in line with previous research evidence.^30–32^ Nonetheless, TNBC and the high-risk group showed highly overlapping IDFS and DRFS curves, indicating similar risk of recurrence. However, the observed crossover in Kaplan-Meier curves suggests distinct recurrence kinetics between TNBC and the high-risk group. TNBC initially shows better IDFS and DRFS, likely due to the benefits of aggressive upfront chemotherapy and a subset of patients achieving pathological complete response. However, after the first year, the recurrence risk rises for TNBC, aligning with its well-documented pattern of early relapses. In contrast, the high-risk group has a more sustained risk over time, leading to a crossover. By year 4, the curves converge, reflecting TNBC’s risk plateau and the high-risk group’s continued recurrence burden. According to SEER statistics, all TNBC patients, irrespective of disease stage, have a 5-year OS rate of 77.1%. With localized disease, the 5-year OS rate is 91.3% but drops to 65.8% in patients with regional disease.^42^ The 5-year OS rate for the TNBC group in the current study, comprising stages IA-IIIC, was 89.4%. A Finnish RWE study reported a lower 5-year OS rate of 74.9% for the early TNBC population (n=352) between 2005 and 2018.^8^ The difference may be due to the different study time periods and differences in treatment practices between countries. Because TNBC tumors lack ER, PR, and HER2 expression, they are not sensitive to ET or HER2–targeted treatment, and standardized TNBC treatment regimens are still lacking. Therefore, there is an urgent need to identify molecular, therapeutically relevant targets.

As registry-based studies do in general, this study may have limitations associated with the completeness and accuracy of the data. According to the Annual Report (2021), the coverage rate of the DBCG quality database in relation to Danish Pathology Register at a nationwide level is very high—98.4% (95% CI: 98.1%-98.8%).^48^ There are several validation studies that show a strong agreement between DBCG registry data and medical records, especially for the patient, tumor, and treatment variables.^47,49,50^ In contrast, underreporting of BC recurrence has been observed and, thus, estimates of the absolute risk of recurrence might be downwardly biased.^46,49,50^ This study includes a representative sample of EBC patients in Denmark, where all permanent residents have access to publicly funded health care. Therefore, healthcare accessibility, socioeconomic status, and private health insurance coverage have not introduced selection bias. Clinical management of HR+, HER2– EBC is highly similar across Europe and US; however, adherence rates in US might be lower due to access and cost barriers. Still, results should be generalized with caution, as treatment and recording practices may vary between countries.

## CONCLUSIONS

These real-world data show that about 13.0% of the HR+, HER2– EBC patient population has a high risk of recurrence. A total of 26.1% of patients in the high-risk group—as well as 27.0% of the TNBC patients—experienced a recurrence event within 5 years after surgery (in contrast to 13.9% in the low/moderate-risk group). In line with previous evidence, these results underline that there is a substantial group of patients with a clear unmet need for more effective adjuvant treatments. Knowing the size of the high-risk group may help in planning local treatment strategies and allocating healthcare resources. These patients are likely to benefit from novel treatment strategies targeted for patients with a high risk of recurrence.

### Ethics Approval and Consent to Participate

This observational study was approved by Statistics Denmark (708444), the Danish National Board of Health (FSEID-00005829), and the National Clinical Registries Program (RKKP) (DBCG-2021-09-29). The study was performed in accordance with the Declaration of Helsinki and in compliance with applicable national laws. The study was based on existing data and no interventions were performed. Ethical approval or informed consent were not required.

### Data Availability

The data sets generated and/or analyzed during the current study are not publicly available due to being subject to a project-specific data permit but are available from the corresponding author on reasonable request.

## Supplementary Material

Online Supplementary Material
