# Supplementary material for: Size and Treatment Outcomes of HR+, HER2- Early Breast Cancer Population With High Risk of Recurrence: A Real-World Cohort Study With Danish Breast Cancer Cooperative Group Registry Data
Source: J Health Econ Outcomes Res. 2025 Jun 24;12(1):252–60. doi: 10.36469/001c.137277 (PMC12201145; doi:10.36469/001c.137277)

Journal of Health Economics and Outcomes Research

Online Supplementary Material

Size and Treatment Outcomes of HR+, HER2- Early Breast Cancer Population With High Risk of Recurrence: A Real-World Cohort Study With Danish Breast Cancer Cooperative Group Registry Data. *JHEOR*. 2025;12(1):252-260. doi:10.36469/jheor.2025.137277

Table S1: Cohort Attrition and Subgroup Flow

Figure S1: Multivariate Cox Model Results (Hazard Ratio [CI]) of Factors Associated With Invasive Disease-Free Survival in the High-Risk Patient Group

Figure S2: Multivariate Cox Model Results (Hazard Ratio [CI]) of Factors Associated With Distant Recurrence-Free Survival in the High-Risk Patient Group

Figure S3: Multivariate Cox Model Results (Hazard Ratio [CI]) of Factors Associated With Overall Survival in the High-Risk Patient Group

This supplementary material has been provided by the authors to give readers additional information about their work.

This is an open-access article distributed under the terms of the Creative Commons Attribution 4.0 International License (CCBY-4.0). View this license's legal deed at http://creativecommons.org/licenses/by/4.0 and legal code at http://creativecommons.org/licenses/by/4.0/legalcode for more information.

Table S1. Cohort Attrition and Subgroup Flow

Level	Population	N	Notes
0	Base population	51 165	BC diagnosis in DBCG, Jan. 1, 2010–Dec. 31, 2019
1	Study cohort	33 631	All inclusion and exclusion criteria applied (see Methods)
1.1	EBC (stage IA-IIIC)	30 482	Excluded 3149 (from 33 631) with TNM 0 or unknown.
1.1.1	HR+, HER2– EBC	23 813	Excluded 6669 (from 30 482) with HR- or HER2+ or either with unknown status (n = 583)
1.1.1.1	HR+, HER2–, node positive EBC (stage IB-IIIC)	8406	Excluded 15 407 (from 23 813) with node negative or unknown status (n = 332)
1.1.1.1.1	High-risk	3098	As defined in Methods
1.1.1.1.2	Low/moderate risk	5308	As defined in Methods
1.1.2	TNBC	2520	Excluded 27 962 (from 30 482) with HR+ or HER2+ or either with unknown status (n = 583)

Abbreviations: DBCG, Danish Breast Cancer Cooperative Group; EBC, early breast cancer; HER2, human epidermal growth factor; HR, hormone receptor; TNBC, triple-negative breast cancer.

Figure S1. Multivariate Cox Model Results (Hazard Ratio [CI]) of Factors Associated With Invasive Disease-Free Survival in the High-Risk Patient Group

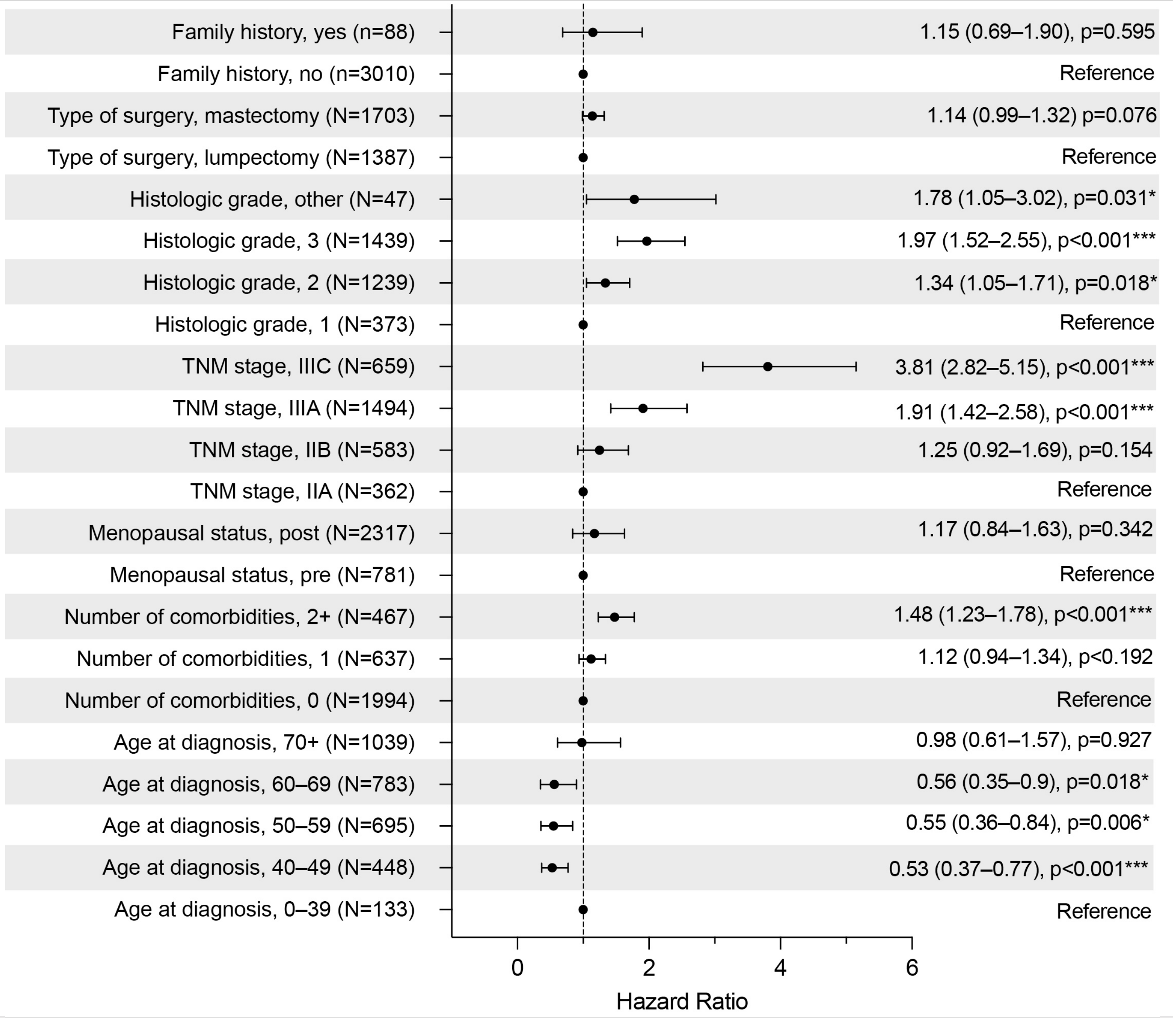

Figure S2. Multivariate Cox Model Results (Hazard Ratio [CI]) of Factors Associated With Distant Recurrence-Free Survival in the High-Risk Patient Group

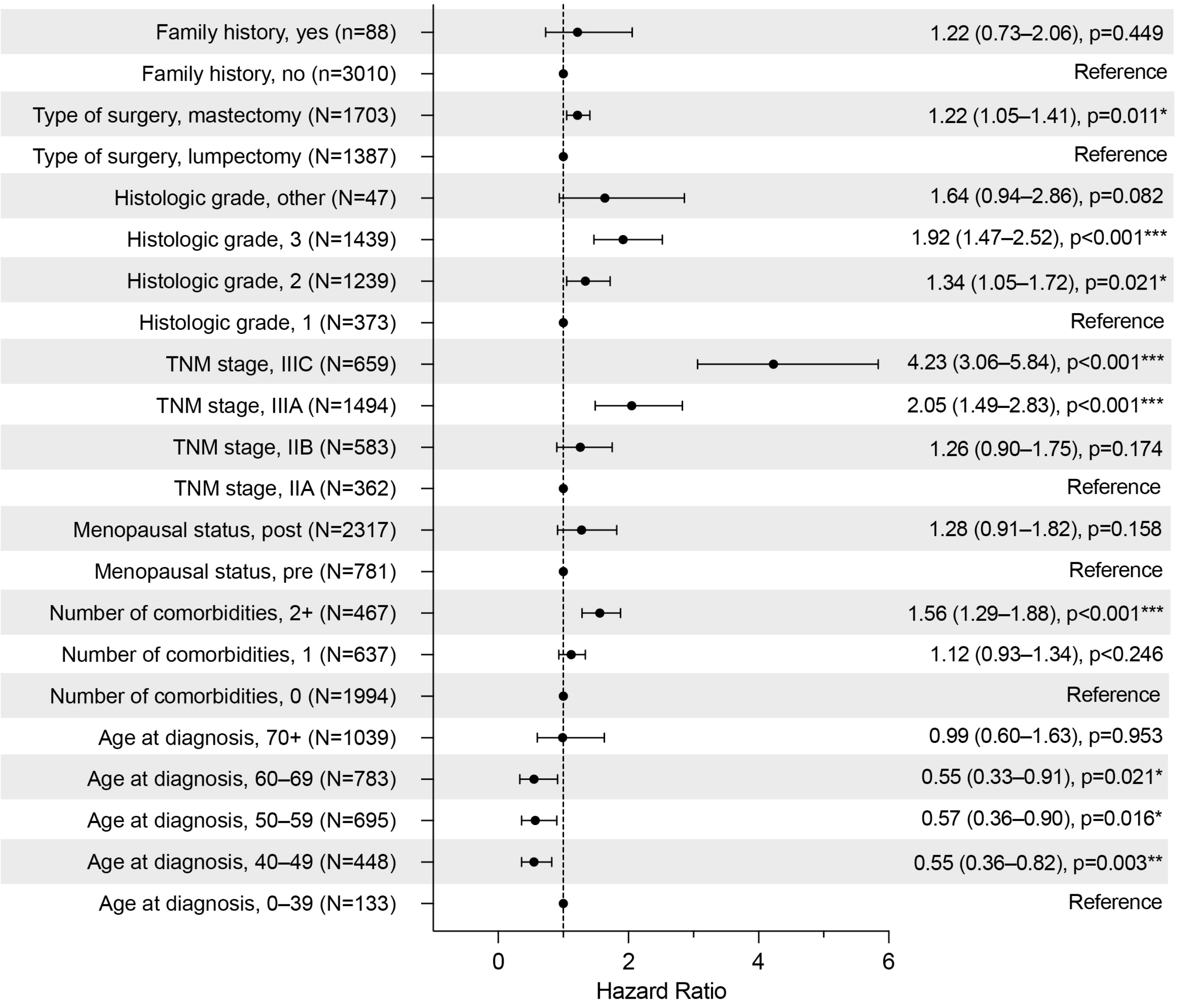

Figure S3. Multivariate Cox Model Results (Hazard Ratio [CI]) of Factors Associated With Overall Survival in the High-Risk Patient Group

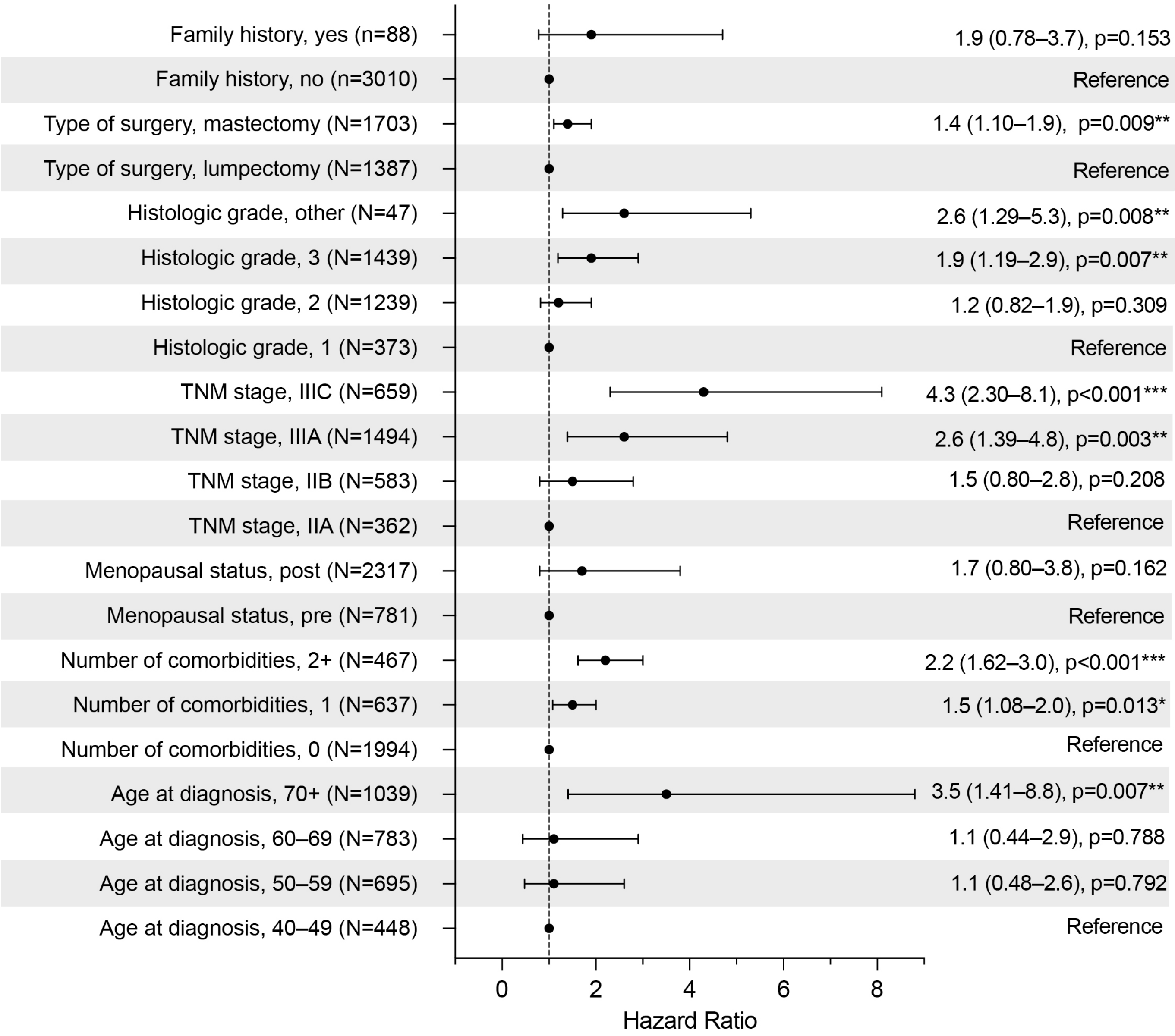

Supplement: Online Supplementary Material [file jheor_2025_12_1_137277_290677.pdf]
